# Prognostic Modeling of Patients Undergoing Surgery Alone for Esophageal Squamous Cell Carcinoma: A Histopathological and Computed Tomography Based Quantitative Analysis

**DOI:** 10.3389/fonc.2021.565755

**Published:** 2021-04-12

**Authors:** Lei-Lei Wu, Jin-Long Wang, Wei Huang, Xuan Liu, Yang-Yu Huang, Jing Zeng, Chun-Yan Cui, Jia-Bin Lu, Peng Lin, Hao Long, Lan-Jun Zhang, Jun Wei, Yao Lu, Guo-Wei Ma

**Affiliations:** ^1^ Sun Yat-sen University Cancer Center, State Key Laboratory of Oncology in South China, Collaborative Innovation Center for Cancer Medicine, Sun Yat-sen University, Guangzhou, China; ^2^ School of Data and Computer Science, Sun Yat-sen University, Guangzhou, China; ^3^ Department of Radiology, University of Michigan, Ann Arbor, MI, United States

**Keywords:** quantitative analysis, esophageal cancer, prognosis, medical images, survival model

## Abstract

**Objective:**

To evaluate the effectiveness of a novel computerized quantitative analysis based on histopathological and computed tomography (CT) images for predicting the postoperative prognosis of esophageal squamous cell carcinoma (ESCC) patients.

**Methods:**

We retrospectively reviewed the medical records of 153 ESCC patients who underwent esophagectomy alone and quantitatively analyzed digital histological specimens and diagnostic CT images. We cut pathological images (6000 × 6000) into 50 × 50 patches; each patient had 14,400 patches. Cluster analysis was used to process these patches. We used the pathological clusters to all patches ratio (PCPR) of each case for pathological features and we obtained 20 PCPR quantitative features. Totally, 125 computerized quantitative (20 PCPR and 105 CT) features were extracted. We used a recursive feature elimination approach to select features. A Cox hazard model with L1 penalization was used for prognostic indexing. We compared the following prognostic models: Model A: clinical features; Model B: quantitative CT and clinical features; Model C: quantitative histopathological and clinical features; and Model D: combined information of clinical, CT, and histopathology. Indices of concordance (C-index) and leave-one-out cross-validation (LOOCV) were used to assess prognostic model accuracy.

**Results:**

Five PCPR and eight CT features were treated as significant indicators in ESCC prognosis. C-indices adjusted for LOOCV were comparable among four models, 0.596 (Model A) vs. 0.658 (Model B) vs. 0.651 (Model C), and improved to 0.711with Model D combining information of clinical, CT, and histopathology (all p<0.05). Using Model D, we stratified patients into low- and high-risk groups. The 3-year overall survival rates of low- and high-risk patients were 38.0% and 25.0%, respectively (p<0.001).

**Conclusion:**

Quantitative prognostic modeling using a combination of clinical data, histopathological, and CT images can stratify ESCC patients with surgery alone into high-risk and low-risk groups.

## Introduction

Esophageal cancer (EC) is the seventh most common cancer and the sixth leading cause of cancer-related mortality globally (1). The main histological subtypes of EC include esophageal squamous cell carcinoma (ESCC) and esophageal adenocarcinoma (EA) (1–3). ESCC accounts for approximately 79% of ECs worldwide, and in China, more than 90% of all ECs are ESCCs (4, 5). Esophagectomy is a routine treatment for ESCC in current clinical practice. However, the 5-year overall survival rate of ESCC is only 20–40% (6) and the postoperative care of patients with ESCC remains challenging. Therefore, the current postoperative care protocol for the management of patients with ESCC needs to be optimized to improve their life expectancy.

An important aspect of postoperative care is the development of tools for disease staging and prognosis prediction, where the goal is to assist physicians in the pre-selection of patients with poor prognosis who may have a high risk of metastasis and/or recurrence. If successful, such tools will improve the curative rates for ESCC. The available studies suggest that radiological image analysis plays an important role in the prognosis of patients with malignant tumors. Previous studies demonstrated the role of quantitative analysis of computed tomography (CT) and pathological images in prognosis prediction for lung cancer (7–9), hypopharyngeal cancer (10), colorectal cancer (11), and other such cancers (12–14). In very few studies based on immunohistochemical staining for the diagnosis of the condition and analysis of disease prognosis (15–19), pathological hematoxylin-eosin (HE) staining images were used for direct feature extraction and prognosis analysis (20–22). This is because the delineation of pathological images is time-consuming, and delineating tumor cells and computerizing the morphology of tumor cells. Recently, for pathological quantitative analysis, some studies used deep learning systems to elevate the accuracy of diagnosis in prostate cancer and improve the predictive ability for the prognosis of malignant tumors (21, 22).

**Figure 1:**
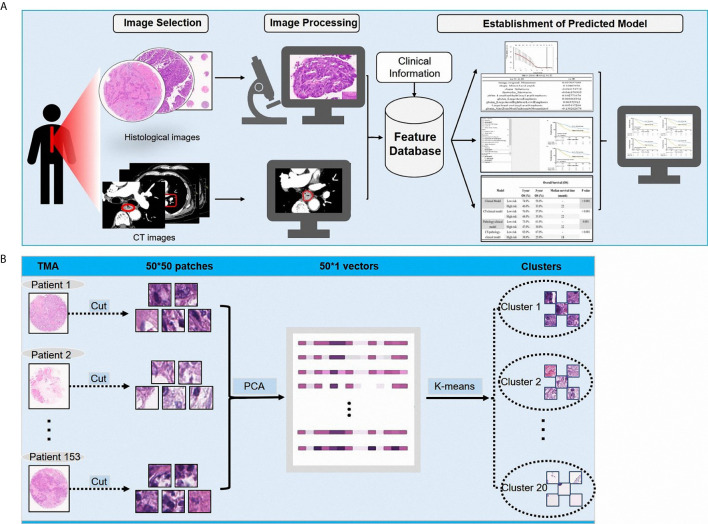
**(A)** The schematic diagram showing the study design; **(B)** The processing of pathological images.

In this study, we aimed to develop a prognostic prediction model of ESCC using CT, pathological, and clinical data. We extracted the features from the diagnostic CT images and pathological images of patients with ESCC, from which the useful information was extracted using a clustering analysis method. This is the novel report describing the development of a prognostic prediction model using the clustering analysis method for extracting useful artifacts from collected pathological data. The schematic diagram of the model is illustrated in **Figure 1A**.

## Methods

### Study Cohort

The Ethics Committee of Sun Yat-sen University Cancer Center approved the study’s protocol (Approval No: GZR2019-161), the requirement of informed consent was waived. The eligible cohort included ESCC patients with pathological and CT findings who underwent esophagectomy at the Department of Thoracic Surgery of a university cancer center in China between January 2014 and August 2015. Patients were excluded if they: (1) had received neoadjuvant chemotherapy, radiotherapy, or immunotherapy; (2) had been diagnosed with another malignancy currently or in the past; (3) had incomplete resection or residual tumor cells; (4) had died due to postoperative complications; (5) had tumors located at the esophagogastric junction or cervical esophagus; or (6) had other histological subtypes of esophageal cancer besides ESCC. According to the patient records, we translated the pathological staging according to the 8^th^ edition of the American Joint Committee on Cancer (AJCC) Staging Manual. Under our study’s protocol, a total of 153 patients were retrospectively analyzed.

### Surgery

Popular esophagectomy procedures for ESCC include the Sweet (left thoracotomy and diaphragm incision), the McKeown (right thoracotomy, laparotomy, and neck incision), and the Ivor Lewis (laparotomy and right thoracotomy). In this cohort of patients, thoracoabdominal lymphadenectomy was performed as the standard procedure. During the operation, the median and mean the number of lymph nodes dissected was 18.0 and 20.54, respectively.

### Follow-Up

We recommended patients visit the outpatient department for follow-up examinations every 3 months for the first 2 years, then every 6 months for the next 3 years, and annually thereafter. Follow-up examinations consisted of recording history, physical examination, chest radiography, cervical ultrasonography, abdominal ultrasonography, barium esophagography, and neck-abdomen CT scans. If necessary, patients also underwent positron emission tomography-CT and/or endoscopy examinations. In the study cohort, the median follow-up time was 28.0 months.

### Tissue Microarray (TMA)

The use of the ESCC TMA was authorized by the ethics committee of Sun Yat-sen University Cancer Center (Approval No: GZR2019-161). The pathologist first reviewed the pathological HE staining slides and sketched the most abundant areas of the malignant tumor. Then, the corresponding positions of the sketched areas were punched into the paraffin tissue. HE staining was performed to form a tissue microarray. We performed whole-slide microarray imaging to obtain an integral pathological image.

### Quantitative Imaging Analysis

The radiologist sketched the 3D regions of interest (ROIs) of all images obtained on CT using an open-source software, Amira 5.4 (Thermo Fisher Scientific, Inc., Waltham, Massachusetts, USA). We extracted CT features from 3D CT ROIs; 105 CT quantitative features were extracted by using Pyradiomics 2.2.0 (in Python software 3.6; https://www.python.org).

Pathological images represent biological information at a different scale from that of CT images. Previous studies focused more on gray level features, geometric features, and texture features extracted from CT images. For pathological images, image patterns represent the internal characteristics and the external distribution of cells particularly related to clinical outcomes. Given the cellular level of the pathological scale, to utilize the histological information and further explore the pathological patterns, we cut the images (6000 **×** 6000) of each patient into 50 **×** 50 patches, and obtained a total of 14,400 patches for every patient. This size of patches was convenient to observe the pattern of ESCCs and to further analyze the medical images. To determine the distribution of these patches in patients with ESCC, we used a cluster analysis method. A principal component analysis (PCA) and a K-means algorithm were used to divide all patches into 20 clusters, with each cluster representing one kind of cell-scale patch. In the pathological slide of each patient, the patch ratio of each cluster represented the ratio of the kind of cells. We used the pathological patches cluster ratio (PPCR) for pathological features and we obtained 20 PPCR quantitative features. **Figure 1A** illustrates the image analysis pipeline. **Figure 1B** shows an example of a cell level patch from the whole microarray slide.

### Statistical Analysis

Statistical analyses were performed using SPSS Statistics 25.0 software (IBM SPSS, Inc., Chicago, IL, USA) and R version 3.6.2 (https://www.r-project.org/). Hazard ratios (HR) with 95% confidence intervals (95% CIs) were calculated by Cox univariate and multivariate regression analyses methods. Heatmaps were used to present with the distribution of 20 PPCR features in 153 patients with ESCC after cluster analysis. Recursive feature elimination (RFE) was used to select features from clinical features, CT features, and pathological features (23). The factors with a two-sided p-value of <0.2 were incorporated into the constructing model. We used an L1-norm penalized Cox (LASSO-Cox) model, which could effectively prevent overfitting in the multivariate survival analysis, and found the best-penalized coefficient each time. The clinical survival model contained four features, the CT survival model contained eight CT quantitative features, the pathological survival model contained five quantitative PPCR features, and the CT-pathology-clinical model used all 17 quantitative features.

We built four LASSO-Cox models, combined all features into a survival score (SC), and divided patients into two groups (high-risk group and low-risk group) according to the median value of the SC. Harrell’s C index was generated for discrimination of the prognostic model. To verify the stability of the small sample model, we performed leave-one-out cross-validations (LOOCV). According to the LOOCV results, we found that the improvement was significant when we combined the medical image features with clinical features to build a LASSO-Cox model. Kaplan–Meier analysis and log-rank tests were used to compare survival curves between groups. A two-sided p-value of <0.05 was considered statistically significant. Cases were censored at death or the end of follow-up. The selection of overall survival as a primary clinical endpoint was considered most clinically relevant.

## Results

### Clinical Features

The clinical characteristics of patients in the cohort are listed in **Table 1**. Of the 153 enrolled patients, 123 (80.4%) were males and 30 (19.6%) were females, with an age range from 41 years to 84 years (median, 61 years). Tumor differentiation was well-moderated in most cases (N = 130, 85.0%) and poor in the rest of the patients (N = 23, 15.0%). The most common tumor, node, metastasis (TNM) stages of patients were IB (N = 56, 36.6%) and IIIA (N = 64, 41.8%). Ninety-one patients (59.5%) had a smoking history and 64 patients (41.8%) had a history of alcohol consumption. The 1- and 3-year overall survival rates were 60.0% and 46.0%, respectively. The median time from the operation to the last censoring date was 28.0 months, and the mean time was 31.84 months.

**Table 1:** The characteristic of clinical information in patients with ESCC.

Variable	No.	%
**Sex**		
Male	123	80.4
Female	30	19.6
**Age (years)**		
≤65	111	72.5
>65	42	27.5
**Smoking status**		
No	62	40.5
Yes	91	59.5
**Drinking status**		
No	89	58.2
Yes	64	41.8
**Tumor location**		
Upper	3	2.0
Middle	70	45.8
Lower	80	52.3
**Tumor length** (cm)		
≤5	115	75.2
>5	38	24.8
**Differentiation**		
Well–moderate	130	85.0
Poor	23	15.0
**TNM stage**		
IB	5	3.3
IIA	56	36.6
IIB	8	5.2
IIIA	8	5.2
IIIB	64	41.8
IV	12	7.8
**T stage**		
T1	10	6.5
T2	32	21.0
T3	107	69.9
T4a	4	2.6
**N stage**		
N0	69	45.1
N1	45	29.4
N2	28	18.3
N3	11	7.2
**Nerve invasion**		
No	81	52.9
Yes	72	47.1
**Vascular invasion**		
No	147	96.1
Yes	6	3.9
**Amount of bleeding**		
≤ 400ml	152	99.3
> 400ml	1	0.7
**CRP**		
≤ 5mg/ml	106	69.3
> 5mg/ml	47	30.7
**AGR**		
≤ 1.5	96	62.7
> 1.5	57	37.3
**Tumor thrombus**		
No	100	65.4
Yes	53	34.5
**Adjuvant therapy**		
No	146	95.4
Chemotherapy	3	2.0
Radiotherapy	4	2.6

### Extraction and Selection of Features of Images

**Figure 2:**
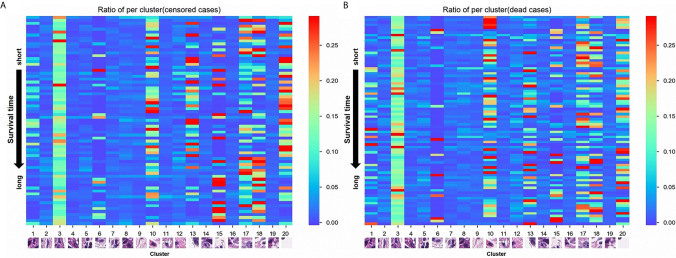
**(A)** The distribution of 20 pathological patches cluster ratio (PPCR) features in alive patients with esophageal squamous cell cancer (ESCC); **(B)** The distribution of 20 PPCR features in dead patients with ESCC.

**Figure 3:**
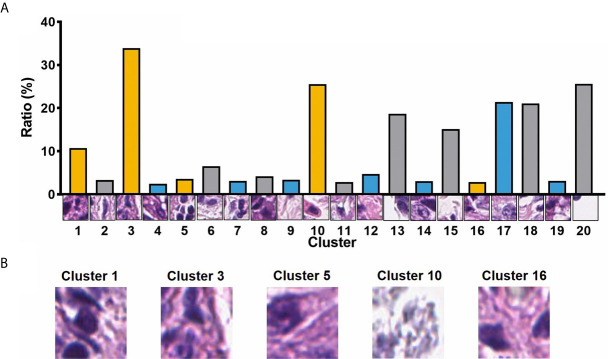
**(A)** The ratio of 20 PPCR features to all patches; **(B)** visualization of image patches influencing outcomes of patients.

A total of 105 textural features (six types of radiomics features, **Table 2**) were extracted from CT images from the 153 patients in the cohort. Following the extraction of pathological features from the pathological TMA images, we got 20 PPCR features. In the heatmap, we could observe the distribution of all clusters in 153 patients with ESCC, and Cluster 10 presented with a color gradient process (**Figure 2**). We found that Cluster 3 had the highest ratio in all patches (**Figure 3A**). Then we used RFE to select a total of four clinical features, and 13 medical image features which included five PPCR features and eight CT features (**Table 3** and **Figure 3B**).

**Table 2:** Six types of radiomics features and the number of them.

Feature class	Count
Shape 3D	16
First Order Statistics	19
Gray Level Co-occurrence Matrix	24
Gray Level Run Length Matrix	16
Gray Level Size Zone Matrix	16
Gray Level Dependence Matrix	14

**Table 3:** Selected features from medical images and clinical information by RFE.

Features	Statistic		
	HR	P value	95% CI
**CT features**			
original_shape_LeastAxisLength	0.41	0.04	0.02-0.81
original_shape_Maximum2DDiameterColumn	1.07	<0.005	0.46-1.67
original_shape_Maximum3DDiameter	1.47	<0.005	0.75-2.18
original_shape_Sphericity	0.22	0.17	0.09-0.53
original_glszm_GrayLevelNonUniformityNormalized	0.38	0.15	0.14-0.91
original_glszm_LargeAreaEmphasis	0.34	0.01	0.08-0.6
original_glszm_ZoneEntropy	0.85	<0.005	0.31-1.39
original_ngtdm_Contrast	0.3	0.03	0.03-0.57
**Pathological features**			
Cluster 1	0.01	0.01	0-0.01
Cluster 3	0	0.15	0-0.01
Cluster 5	0.05	0.01	0.01-0.08
Cluster 10	0.01	0.01	0-0.01
Cluster 16	0.04	0.06	0-0.09
**Clinical features**			
Drinking History	0.32	0.18	0.15-0.79
CRP	0.01	0.01	0-0.02
AGR	1.07	0.05	0.01-2.13
TNM stage	0.14	0.08	0.02-0.3

### Construction of Survival Prediction Model

After constructing the survival models to predict the prognosis of ESCC and the determination of the best-penalized coefficients with the LASSO-Cox model, four survival models were created. The clinical survival model included the TNM stage, the concentration of C-reactive protein (CRP), the albumin-globulin ratio (AGR), and the history of alcohol consumption. The CT-clinical survival model contained eight CT features and four clinical features. The pathology-clinical survival model contained five PPCR features and four clinical variates. The CT-pathology-clinical model used all 17 features. The LOOCV-based C-indexes for the clinical survival model, CT-clinical survival model, pathology-clinical model, and CT-pathology-clinical survival model were 0.596, 0.658, 0.651, and 0.711, respectively. The predictive ability of the CT-pathology-clinical survival model was better than others (all P < 0.05). We found that the improvement was significant when we combined the pathological features with CT features to build a LASSO-Cox model.

### Stratified Effect of Survival Models

**Figure 4:**
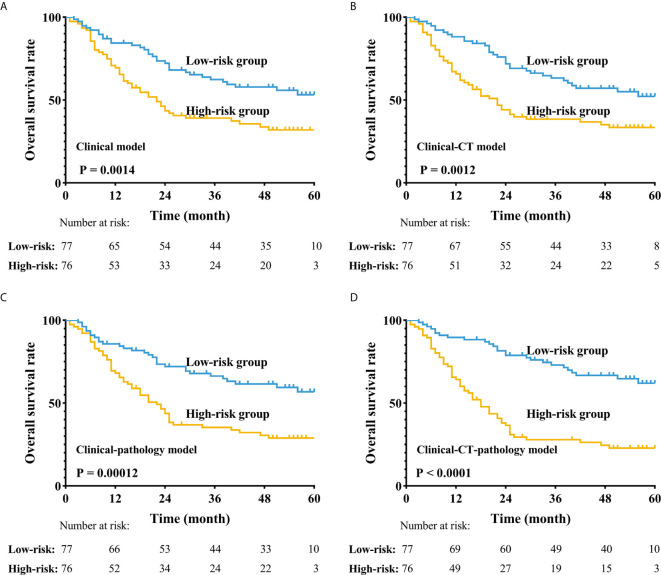
Application of the four different prognostic models to refine the assessment of risk in ESCC. **(A–D)** Kaplan-Meier survival estimates for a group of patients with ESCC from the whole cohorts and the subgroups predicted to have either a high or a low probability of mortality.

After combining all features into a survival score (SC) as a new feature and classifying the patients into the high-risk and low-risk groups according to the median SC, we found that the stratification improved the CT-pathology-clinical survival model (**Figure 4**, P <0.001). In the CT-pathology-clinical survival model, 1- and 3-year overall survival rates were 38.0% vs. 82.0% and 25.0% vs. 67.0% in the high-risk and low-risk groups, respectively (P <0.001; **Table 4** and **Figure 4**).

**Table 4:** Overall survival of patients with ESCC in every model.

Model		Overall Survival (OS)			
		1-year OS (%)	3-year OS (%)	Median survival time (month)	P value
Clinical Model	Low risk	74.0%	58.0%	–	<0.001
	High risk	46.0%	33.0%	22	
CT-clinical model	Low risk	76.0%	57.0%	–	<0.001
	High risk	44.0%	35.0%	22	
Pathology-clinical model	Low risk	73.0%	61.0%	–	0.001
	High risk	47.0%	30.0%	22	
CT-pathology-clinical model	Low risk	82.0%	67.0%	–	<0.001
	High risk	38.0%	25.0%	18	

## Discussion

For analyzing the risks of metastases or recurrence in a cancer patient, the more accurate application of quantitative analysis and correlating it with the knowledge of the individual’s prognostic situation can help identify those with a high risk of metastasis and/or recurrence. Radiomic features of the tumor not recognizable by physicians’ observations can provide information on both the tumor and its microenvironment. In previous studies, deep learning of radiomics has shown unique value in diagnosis and prognosis in the assessment of liver fibrosis in chronic hepatitis B (24), access to tumor-infiltrating CD8 cells, and responses to anti-PD-1 or anti-PD-L1 immunotherapy (25) that could predict lymph node metastasis in ESCC and colorectal cancer (26, 27). CT texture features may be associated with diagnosis and prognosis in malignant tumors, including lung cancer (7, 9, 28–30). In the quantitative analysis of pathological images, deep learning identified adenocarcinoma or squamous cell carcinoma through the pathological image features of lung cancer that could predict the prognosis of cancer, including colorectal cancer and glioma (11, 31–33). In this study, radiologists sketched the ROIs and the location of ?A3B2 twb 0.24w?>tumors in esophageal cancer on CT, and pathologists reviewed the HE-stained images and labeled the tumor-enriched areas, then perforated the corresponding wax blocks to create the TMAs. Essentially, most previous studies in pathology that focused on studying a single cell, nuclei, etc., were limited to paraffin tissue sections, which led to changes in the nucleus and imaging results. While studying single cells, it is difficult to fully reflect the true state of the tumor. Therefore, this study aimed to reflect on as many of the features of the tumor on the pathological slides as possible. Therefore, we used whole microarray slide imaging technology to digitize the pathological images, which we could conveniently process by cutting the original picture of every patient into 50 **×** 50 size patches from 6000 **×** 6000. Then, each patient had a total of 14,400 patches. In addition, we obtained 20 PPCR features after cluster analysis. Eventually, there were five PPCR features reserved. The heatmap showed that Cluster 10 had a color gradient process (**Figure 2**). This study innovatively used cluster analysis to extract pathological features. Using this method of cluster analysis, we processed a large number of pathological images that were convenient and reduced the work of the pathologist; moreover, the features could be automatically identified and sketched by the computer. It is worth mentioning that, as far as we know, this study is the first to use recursive feature elimination (RFE) as a statistical algorithm in the field of medical imaging. The features selected by this algorithm helped us to optimize the model’s predictive ability.

As we all know that neoadjuvant therapy (such as chemotherapy, radiotherapy and chemoradiotherapy) is necessary for local advanced ESCC patients to improve survival outcomes (34, 35). For this research, we want to discuss the importance of analyses to diagnostic CT and postoperative histological slides more. Therefore, we excluded the patients who received neoadjuvant therapy to reduce the influence of neoadjuvant therapy on histological images. However, it’s also important for patients with advanced stage to receive adjuvant therapy to get a survival benefit. Regrettably, given the flaws of a retrospective study, we unable to obtain more detailed information about adjuvant therapy. Because the part of ESCC patients live far from our cancer center, they may receive adjuvant therapy in the local hospital, but we unable to record this part of the information. Thus, we try to prospectively recruit the ESCC patients to further study the impact of quantitative features to CT and pathological images on prognosis.

We built a clinical survival model based on four features, yielding a C-index adjusted for LOOCV of 0.596, indicating a difference between low-risk and high-risk groups (**Figure 4A**). We found that the CT-clinical model was very effective in distinguishing patient prognoses (**Figure 4B**) and stratified postoperative patients who were prone to recurrence and metastasis. During pathological image processing, we adopted an innovative cluster analysis method, and our pathological-clinical model based on quantitative features was much improved compared to the initial clinical model (P <0.05). The reason that the CT-clinical model was better than the pathology-clinical model might be that a single pathological slice does not fully reflect the biological characteristics of the tumor; however, the CT model is based on 3D dimensional features of ESCC. Finally, the CT-pathology-clinical model was significantly better at predicting prognosis than the CT-clinical and pathology-clinical models (P <0.001, **Figure 4**), as it is based on CT, clinical, and pathological data.

Our study combined multidisciplinary data to construct a more comprehensive and more individualized prognostic model in ESCC. Previous studies focused on qualitative observation of images (36–38); by contrast, this study focused on quantitative analysis of CT and pathological images. This was more precise compared to quantitative analysis for creating a survival model, and therefore, more accurate for predicting the prognosis of ESCC patients after surgery.

There are some limitations of this study. First, this was a retrospective study with a small number of cases (153 patients). We verified the stability of our small sample model using a LOOCV; however, it remains necessary to increase the sample size and conduct a prospective study to verify our results. In addition, the distribution of each patient in the staging was unbalanced and represented a skewed distribution. Second, pathologists selected tissue enrichment areas to create the TMA; however, information around the tumor was lost. Furthermore, the results of TMA might lack random effect because of artificial selection; in other words, selecting different tumor areas might have a deviation in experimental results. Third, this study only evaluated the primary tumor in images of CT and pathology; indeed, the status of lymph nodes was also a key indicator to predict the prognosis of ESCC, therefore, further quantitative analysis of medical images with lymph nodes in CT and pathology is needed. Eventually, the results of this study can only reflect the prognosis of patients with postoperative ESCC. Treatment decisions remain the responsibility of clinicians and need to be based on the individual patient’s situation.

In conclusion, quantitative prognostic modeling using a combination of clinical, histopathological, and diagnostic CT data can stratify ESCC patients with surgery alone into high-risk and low-risk groups, improving the prediction of the prognosis of postoperative patients. This comprehensive model included individual information reflecting more accurate conditions of ESCC, assisting precise treatment.

## Data Availability Statement

Researchers interested in this study may contact the authors to obtain the clinical data of all 153 patients. We have uploaded the data of this study in the Research Data Deposit of SYSUCC, and its number was RDDB2021001083 (http://www.researchdata.org.cn/).

## Ethics Statement

The studies involving human participants were reviewed and approved by ethics committee of Sun Yat-sen University Cancer Center (Approval No: GZR2019-161). Written informed consent for participation was not required for this study in accordance with the national legislation and the institutional requirements.

## Author Contributions

Conception and design: G-WM, YL, JW. Administrative support: PL, HL, L-JZ. Provision of study materials or patients: PL, HL, L-JZ, G-WM. Collection and assembly of data: G-WM, YL, JW, L-LW, WH, XL, J-LW, J-BL, C-YC, JZ, Y-YH. Data analysis and interpretation: G-WM, YL, JW, L-LW, WH, J-LW. Manuscript writing: G-WM, YL, JW, L-LW, WH, J-LW. Final approval of manuscript: G-WM, YL JW, L-LW, WH, XL, J-LW, J-BL, C-YC, JZ, Y-YH. Made of TMA: J-BL, WH, JZ.. Selection of tumor region in CT: G-WM, WH, L-LW, XL, C-YC. All authors contributed to the article and approved the submitted version.

## Funding

This work was supported in part by the National Key R&D Program of China under Grant 2018YFC1704206, in part by the National Natural Science Foundation of China under Grant 81971691, Grant 81830052, and Grant U1811461, in part by the Science and Technology Innovative Project of Guangdong Province under Grant 2016B030307003, in part by the Guangdong Esophageal Cancer Institute Science and Technology Program under Grant M201916.

## Conflict of Interest

The authors declare that the research was conducted in the absence of any commercial or financial relationships that could be construed as a potential conflict of interest.
